# Emergence of cognitive priming and structure building from the hierarchical interaction of canonical microcircuit models

**DOI:** 10.1007/s00422-019-00792-y

**Published:** 2019-02-14

**Authors:** Tim Kunze, Jens Haueisen, Thomas R. Knösche

**Affiliations:** 1 Max Planck Institute for Human Cognitive and Brain Sciences, Leipzig, Germany; 2 Institute of Biomedical Engineering and Informatics, Ilmenau University of Technology, Ilmenau, Germany

**Keywords:** Canonical microcircuit, Hierarchical model, Neural computations, Adaptive mechanisms, State-dependent operation, Syntax parsing

## Abstract

The concept of connectionism states that higher cognitive functions emerge from the interaction of many simple elements. Accordingly, research on canonical microcircuits conceptualizes findings on fundamental neuroanatomical circuits as well as recurrent organizational principles of the cerebral cortex and examines the link between architectures and their associated functionality. In this study, we establish minimal canonical microcircuit models as elements of hierarchical processing networks. Based on a combination of descriptive time simulations and explanatory state-space mappings, we show that minimal canonical microcircuits effectively segregate feedforward and feedback information flows and that feedback information conditions basic processing operations in minimal canonical microcircuits. Further, we derive and examine two prototypical meta-circuits of cooperating minimal canonical microcircuits for the neurocognitive problems of priming and structure building. Through the application of these findings to a language network of syntax parsing, this study embodies neurocognitive research on hierarchical communication in light of canonical microcircuits, cell assembly theory, and predictive coding.

**Electronic supplementary material:**

The online version of this article (10.1007/s00422-019-00792-y) contains supplementary material, which is available to authorized users.

## Introduction

Connectionism states that higher cognitive functions emerge from the constructive interaction of a large number of relatively simple and uniform fundamental units. The concept of canonical microcircuits (Douglas and Martin 1991, 2007; Douglas et al. 1989) postulates the existence of such fundamental units in the neocortex, which can be effectively described by a common basic circuitry. This idealized local architecture is supposed to give rise to a limited set of stereotypic functions (Silberberg et al. 2002; Harris and Shepherd 2015), referred to as basic operations (Kunze et al. 2017), universal computational capabilities (Haeusler et al. 2009), or computational primitives (Douglas and Martin 2007). In Kunze et al. (2017), we defined a minimal canonical microcircuit (mCMC) as a system of interacting excitatory and inhibitory neural populations whose structural topology reflects the homogeneity of neocortical matter and whose functional repertoire features the basic operations of signal flow gating and working memory.

Various computational models have been proposed for the cooperation of multiple canonical microcircuits to realize higher cognitive functionality, such as attentional processing (Ardid et al. 2007), predictive coding (Bastos et al. 2012), language processing (Pulvermüller et al. 2014; Wennekers et al. 2006), and visual steering (Heinzle et al. 2007). Extending this research, here we investigate how structure building and priming emerge from the hierarchical interaction of mCMCs.

Cognitive functions correspond to specific neural activation patterns. Structure building refers to the underlying mechanisms of their establishment (Fries 2005; Rolls and Deco 2015; Friston 2008; Spiegler et al. 2016) and is a central element of cell assembly theory (Hebb 1949; Braitenberg 1978; Pulvermüller et al. 2014; Palm et al. 2014). Cell assemblies (sometimes called neuronal assemblies) denote groups of neurons with strong mutual excitatory connections that together represent objects or more abstract entities of thought (Wennekers et al. 2003). The progressive activation of these cell assemblies, called *association,* was proposed to embody cognitive processing. It has been used to describe mechanisms in various cognitive disciplines including perception, memory, decision making, attention, and language tasks (Pulvermüller et al. 2014; Wennekers et al. 2006). Wennekers et al. (2006) formalize three different kinds of cell assembly associations: (i) *auto*-*association*, where cell assemblies project onto themselves to enhance localized activations (that is, attractor states), (ii) *heteroassociations*, where two or more cell assemblies sequentially activate each other, similar to the concept of synfire chains (Abeles 1991), and (iii) *conditioned associations* that extend the spontaneous transitions of heteroassociations by an additional input that is required to gate the activation of cell assemblies and support *selective* structure building (termed synfire graphs). Notably, this formalized functionality is very similar to the basic operations of working memory and signal flow gating, identified in mCMCs (Kunze et al. 2017). In this study, we explicitly identify state-dependent operations in mCMCs.

Priming involves the activation of implicit memory contents by the primer stimulus in a bottom-up fashion, which then influences the processing of the subsequently presented target stimulus in a top-down fashion (Schacter and Buckner 1998; Tulving and Schacter 1990; Kristjansson 2008). It may entail facilitation of perception, increase in attention, and increase in response probability and speed (Kristjansson 2008; Tulving and Schacter 1990). Though considered a non-conscious form of memory, priming operates independently from explicit memory systems. It is difficult to localize and occurs for various sensory modalities and levels of perception (Schacter and Buckner 1998; Kristjansson 2008; Tulving and Schacter 1990). The neural mechanistic underpinnings of this multi-scale processing principle remain elusive, although it is suggested to share some characteristics of top-down attentional guidance (Kristjansson 2008).

Both selective structure building and priming involve some sort of conditioning where prior information narrows down the possibilities of subsequent processing steps and thus predicts them. This fundamental processing principle is known as predictive coding (Mumford 1992; Rao and Ballard 1999). It suggests that the brain is continuously predicting future states based on an internal model of the environment which integrates novel sensory and established conceptual information, conveyed by forward and backward connections, from different levels of the cortical hierarchy (Mumford 1992; Bastos et al. 2012; Friston 2005; Shipp 2016).

In the present study, we systematically investigate the characteristic behavior of mCMCs that are employed in hierarchical networks. We show that a mCMC differentiates between feedforward and feedback input and demonstrate how feedback conditions the availability of basic processing operations. We propose two prototypical meta-circuits of cooperating mCMCs that support priming and structure building. Finally, based on these findings, we extended a previously proposed (Kunze et al. 2017) network model performing syntax parsing during sentence perception, in which hierarchically interacting mCMCs integrate unspecific sensory and conceptual information to yield a specific neural activation pattern.

## Theory and analysis

### Description of the minimal canonical microcircuit model

To formularize a canonical microcircuit, we used a neural mass model (Zetterberg et al. 1978; Jansen and Rit 1995) that has recently been examined for its inherent basic processing operations (Kunze et al. 2017). Importantly, this model emphasized the canonicity of a neural circuit not in the strict reproduction of a cortical column (Haeusler et al. 2009), but in the minimal realization of internal positive and negative feedback loops. In the past, this type of mean-field model already served to elucidate mechanisms in processing systems, such as the description of local steady-state system behaviors (Grimbert and Faugeras 2006; Spiegler et al. 2010; Touboul et al. 2011), inferring neural system architectures from empirical data [dynamic causal modeling (David et al. 2006; Friston et al. 2003)], or a potential realization of predictive coding (Bastos et al. 2012, 2015).

**Fig. 1:**
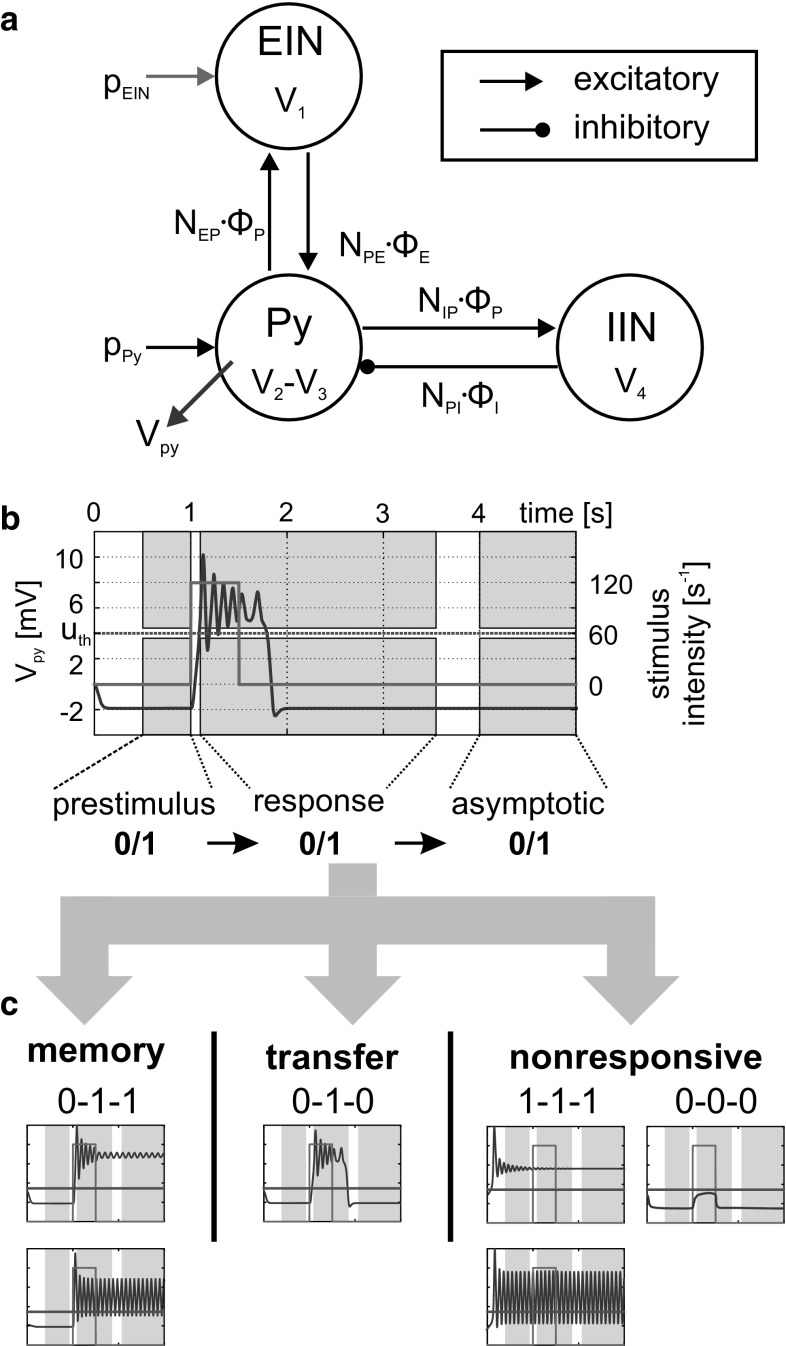
Model architecture and principle analysis approach. **a** The investigated canonical microcircuit model considers three neural masses—pyramidal cells (Py), excitatory interneurons (EIN), and inhibitory interneurons (IIN)—that interact through mean firing rates *φ*(*t*) scaled by connectivity gains *N*_*ab*_. The mean membrane potential *V*_Py_, integrating both positive and negative local feedback from the interneuron populations (*V*_Py_ = *V*_2_ − *V*_3_), serves as output signal. **b** The efferent signal *V*_Py_ (blue line) is compared to a firing threshold *u*_th_ (red line) in three consecutive analysis windows (gray planes) to characterize the response behavior following a transient stimulation (green line). **c** This response behavior was classified into three types: *nonresponsive*, *transfer*, and *memory behavior* (color figure online)

The mCMC model was designed such that it embodies the most important key features of the local cortical circuitry: (1) pyramidal cells providing output to other areas, (2) excitatory and inhibitory feedback to these output neurons, and (3) separate input and output layers. It consists of three neural masses, namely pyramidal cells (Py), excitatory interneurons (EIN),^1^ and inhibitory interneurons (IIN) (Fig. 1a). The Py comprise pyramidal cells in both supragranular (layers II–III) and infragranular (layers V–VI) cortical layers, projecting to other cortical locations. The EIN mainly consist of spiny stellate cells in granular layer IV, but may also include other excitatory cells (pyramidals) that project locally to Py. They are the main target for bottom-up input, thus realizing the separation between input and output populations. Finally, the IIN summarize all inhibitory neurons providing local feedback to both infragranular and supragranular Py. Certainly, this model is a strong simplification. The next sensible extension would be the separation between deep and superficial Py and their associated IIN, which was considered, for example, in Wang and Knösche (2013). Moreover, our model does not feature recurrent feedback loops for the three populations, which certainly do exist (Häusler et al. 2009). Here, however, we used the simplest architecture that has been shown to feature the basic operations of information gating and working memory in our previous study (Kunze et al. 2017).

Each of the three neural masses is described by two state variables: the mean membrane potential *V*(*t*) and the mean firing rate *φ*(*t*). For the synaptic response and the activation functions, we followed the approach by Spiegler et al. (2010), which is based on previous models (Jansen and Rit 1995; Jansen et al. 1993). In each neural mass, the afferent mean firing rate *φ*(*t*), arriving at the dendritic tree of a neural population, is transformed to a respective mean membrane potential *V*(*t*) by convolving the firing rate with a synaptic response kernel *h*_*e*,*i*_(*t*) as in:

1 $$ V_{e,i} \left( t \right) = \varphi \left( t \right)*h_{e,i} \left( t \right), $$

where the index *e* (*i*) denotes the synaptic response kernel of an excitatory (inhibitory) neural mass. The synaptic response kernel is modeled as an alpha-function:

2 $$ h_{e,i} \left( t \right) = \frac{{H_{e,i} }}{{\tau_{e,i} }} \cdot t \cdot \theta \left( t \right) \cdot e^{{{\raise0.7ex\hbox{${ - t}$} \!\mathord{\left/ {\vphantom {{ - t} {\tau_{e,i} }}}\right.\kern-0pt} \!\lower0.7ex\hbox{${\tau_{e,i} }$}}}} , $$

where *θ*(*t*) denotes the Heaviside function, *H*_*e*,*i*_ is the synaptic gain, reflecting number and efficacy of synaptic contacts, and $\tau_{e,i}$ is the characteristic time constant of either excitatory or inhibitory operating neural masses. The mean membrane potential *V*_*c*_(*t*), $c \in \left[ {{\text{P}}, {\text{E}},{\text{I }}} \right]$, of the respective neural masses then depends on the sum of all incoming input components. Using Green’s function, this can be expressed as:

3 $$ D_{e,i} V_{c} = \sum \varphi_{\text{in}} \left( t \right), $$

where *D* is a second-order temporal differential operator:

4 $$ D_{e,i} = \frac{{\tau_{e,i} }}{{H_{e,i} }}\left( {\frac{{{\text{d}}^{2} }}{{{\text{d}}t^{2} }} + \frac{2}{{\tau_{e,i} }} \cdot \frac{\text{d}}{{{\text{d}}t}} + \frac{1}{{\tau_{e,i}^{2} }}} \right), $$

and Eq. (2) represented the Green’s function of this differential operator. The transformation of mean membrane potential to mean firing rates, representing the processes occurring at the axonal hillock of a neuron, is modeled by a sigmoidal activation function, in this case the logistic function:

5 $$ \varphi \left( t \right) = S\left( {V_{c} \left( t \right)} \right) = \frac{{2e_{o} }}{{1 + e^{{r\left( {v_{0} - V_{c} \left( t \right)} \right)}} }}. $$

Here, *e*_0_ represents half of the highest achievable mean firing rate of the respective neural mass, *r* is the maximum slope of the sigmoid function and *v*_0_ denotes the membrane potential for which half of the maximum firing rate was invoked. The system was parameterized with values listed in Table 1.

**Table 1:** Parameterization of the employed neural mass model

Parameter	Value	Unit	Parameter	Value	Unit	Parameter	Value	Unit
*H*_e_	3.25	mV	*N*_EP_	135	–	*r*	0.56	mV^−1^
*H*_i_	22	mV	*N*_PE_	0.8 * N_EP_	–	*v*_0_	6	mV
*τ*_e_	10	ms	*N*_IP_	0.25 * N_EP_	–	*e*_0_	2.5	s^−1^
*τ*_i_	20	ms	*N*_PI_	0.25 * N_EP_	–			

The mean membrane potential of the Py, *V*_Py_(*t*), integrates both positive and negative local feedback from the interneuron populations (*V*_Py_ = *V*_2_ − *V*_3_) and forms the observable signal of the circuit (e.g., by EEG). At the same time, *V*_Py_(*t*) serves as output signal that is transmitted to other coupled mCMCs through the activation function.

Anatomical studies postulated a hierarchical organization of the neocortex with forward connections and backward connections that interlink the different levels of the hierarchy (Felleman and Van Essen 1991). These findings suggest that afferent inputs to a canonical microcircuit target either neurons in the granular layer IV, associated with forward input, or neurons in the agranular layers II/III and V/VI, associated with backward input (Felleman and Van Essen 1991). Following this simplified, though established (Bastos et al. 2012), reflection of hierarchical connections, we associated feedforward signals with input to the excitatory interneurons (*p*_EIN_) and feedback signals with input to the pyramidal cells (*p*_Py_).

According to the scheme depicted in Fig. 1a, the system of governing equations of a single mCMC is:

6 $$ \begin{aligned} & D_{e} \cdot V_{1} = N_{\text{EP}} \cdot \varphi_{\text{Py}} + p_{\text{EIN}} \\ & D_{e} \cdot V_{2} = N_{\text{PE}} \cdot \varphi_{\text{E}} + p_{\text{Py}} \\ & D_{i} \cdot V_{3} = N_{\text{PI}} \cdot \varphi_{\text{I}} \\ & D_{e} \cdot V_{4} = N_{\text{IP}} \cdot \varphi_{\text{P}} \\ & \varphi_{\text{P}} = S\left( {V_{\text{Py}} } \right) = S\left( {V_{2} - V_{3} } \right)  \\ & \varphi_{\text{E}} = S\left( {V_{\text{E}} } \right) = S\left( {V_{1} } \right) \\ & \varphi_{\text{I}} = S\left( {V_{\text{I}} } \right) = S\left( {V_{4} } \right). \\ \end{aligned} $$

The parameters *N*_*ab*_ denote the connectivity gains between the source population *b* and the target population *a*, where $a,b \in \left[ {{\text{P}},{\text{E}},{\text{I}}} \right]$. Heun’s method was employed for the numerical integration of this equation system (6).

### Time simulations, characteristic fingerprints, and bifurcation analysis

The model equations were simulated in a dimensionless form in MATLAB (The MathWorks, Inc., Natick, Massachusetts, USA), and a bifurcation analysis was performed using the numerical continuation tool DDE-BIFTOOL (Engelborghs 2002). Standard methods to compute fixed point curves were used, namely computation of fixed points, derivation of the Jacobian matrix, linearization of the system around the fixed points, and evaluation of the eigenvalues to determine the local stability. We performed time simulations to describe the system behavior, i.e., the formation of neural activity patterns. All state variables were initialized with a zero vector and without external stimulation so that the system consistently resided on the lower branch of the S-shaped fixed point curve in the case of a bistable regime.

**Fig. 2:**
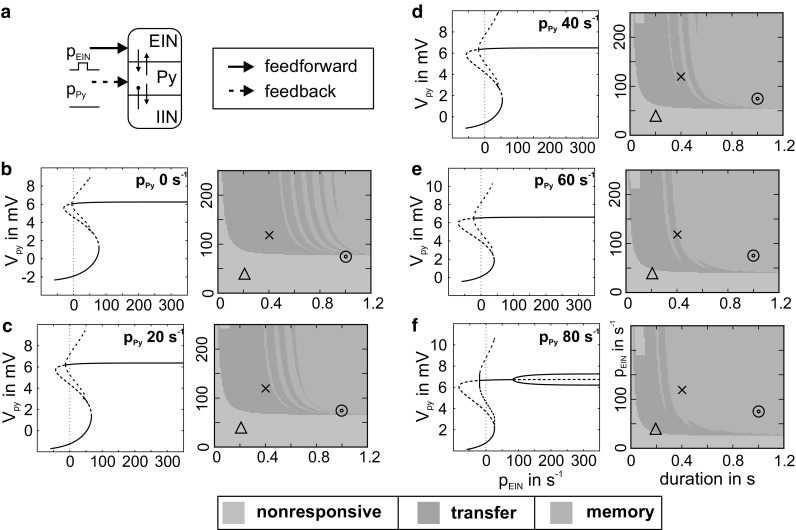
Concomitant stimulation of the mCMC with feedforward and feedback information. **a** The mCMC model simultaneously received transient forward (*p*_EIN_) and constant feedback (*p*_Py_) stimulations. **b**–**f** For increasing levels of constant feedback input (*p*_Py_) the left-hand bifurcation diagrams exhibit stable (solid lines) and unstable (dashed lines) states of the mean membrane potential *V*_Py_ for a range of feedforward input values (*p*_EIN_). These bifurcation plots explain the right-hand characteristic fingerprints that illustrate the consequent response behaviors for transient *p*_EIN_ stimulation. The characteristic fingerprints color-coded the stimulation-induced response behaviors: nonresponsive (green), transfer (gray), and memory behavior (orange). The markers (cross, triangle, and circle) denoted stimuli whose specific response behavior changed according to the level of concomitant feedback input (*p*_Py_) (color figure online)

The information processing in the mCMC model was characterized in terms of the stimulation-induced response behaviors. Following a previous study (Kunze et al. 2017), a neural population was stimulated with a rectangular impulse of defined intensity and duration. The maximum membrane potential of the Py was compared to a firing threshold in three consecutive analysis windows, i.e., prestimulus, immediate response, and asymptotic, see Fig. 1b. Based on the sigmoidal activation function, the firing threshold was defined relative to the maximum firing rate of 5 s^−1^, so that about 25% of the maximum firing rate is reached at the threshold of 4 mV. Three general types of response behavior constitute the basic operations of signal flow gating and working memory: (a) *nonresponsive* for subthreshold transient deflections in *V*_Py_, (b) *transfer* for supra-threshold transient deflections, and (c) *memory* for sustained supra-threshold deflections; see Fig. 1c. The response behaviors were mapped to the stimulus’ properties (namely stimulus duration and intensity) in so-called *characteristic fingerprints* (see Fig. 2, for example), which typified the dynamical response repertoire of the respective parameterization. For further details regarding the mechanisms underlying basic information processing, see Kunze et al. (2017).

## Results

In the following, we first demonstrate that the mCMC model differentiates between afferent feedforward and feedback information and how feedback information conditions the availability of basic processing operations. We then show how the identified facilitative feedback signal gives rise to higher information processing, namely priming and structure building, in prototypical meta-circuits of two cooperating mCMCs. Finally, we apply our findings to a language network for syntax parsing, which integrates syntax predictions and sensory word information in order to establish a syntax prototype.

### Input channels differentiate information flow

The proposed mCMC model features two separate populations to receive feedforward and feedback information, respectively. We stimulated both populations independently with rectangular impulses of defined magnitude and duration and mapped the respective response behaviors (nonresponsive, transfer, and memory; see “Theory and analysis”).

**Fig. 3:**
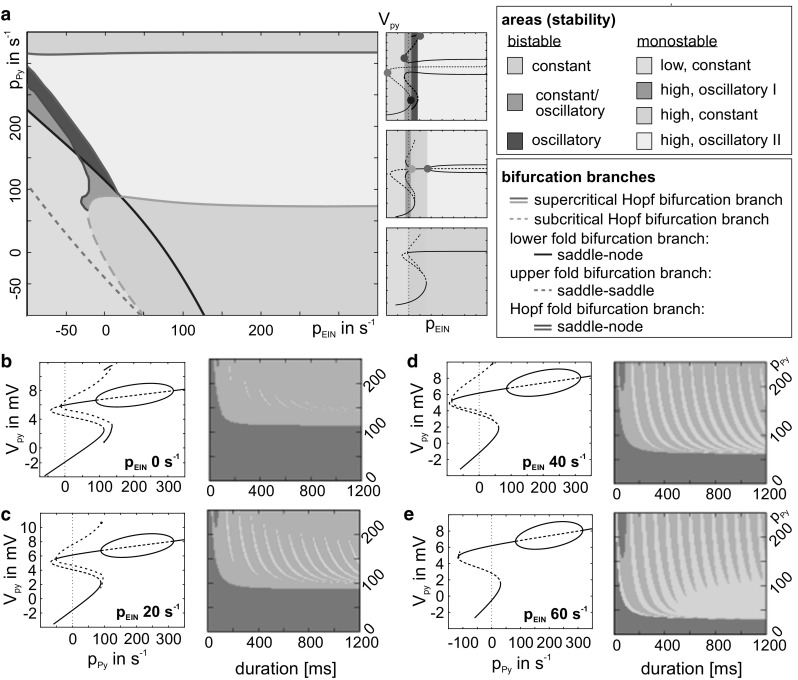
Modulating influence of simultaneous stimulation of EIN and Py. **a** For constant levels of *p*_Py_ and *p*_EIN_ input, the two parameter bifurcation diagram mapped the course of fold and Hopf bifurcations (colored lines). Colored surfaces marked input configurations for which monostable (gray and yellow shadings) or bistable states (green shadings) were present. **b**–**e** Contrary to Fig. 2, we applied increasing levels of constant input to EIN concomitant to a transient Py stimulation. **b** Without modulating input, the characteristic fingerprint (right) shows only sparse traces of memory behavior (orange). **c**–**e** For higher levels of modulating p_EIN_ input, characteristic fingerprints (right) show increased stimulation ranges that induced memory behavior. This is due to the extinction of the separatrix that delimits the memory behavior (left-hand-side state-space projections). In the fingerprints the intensity threshold that separated nonresponsive (green) and transfer (gray) behavior declined, because distance between working point (*p*_Py_ = 0) and the lower fold bifurcation decreased (color figure online)

For stimulation of the Py, we observed large ranges of nonresponsive behavior and only sparse traces of memory behavior embedded in transient behavior (Fig. 3b). In contrast, the same stimuli applied to the EIN evoked nonresponsive behavior for weak stimulations, transfer behavior for strong but brief stimulations, and memory behavior for strong and long stimulations (Fig. 2b) (Kunze et al. 2017). This confirms the driving character of feedforward inputs. Note that the stripe-like memory response behaviors reflect a dependence of the system’s intrinsic oscillation phase on the stimulus’ switch-off time, further explained in Kunze et al. (2017).

These response behaviors were based on dynamics in the state space. For both stimulation targets (EIN and Py) bifurcation and stability analysis yielded an S-shaped fixed point curve whose turning points reflect fold bifurcations, one of saddle–saddle and one of saddle-node type (left panels in Fig. 2b, 3b). Two stable sections of the fixed point curve (one of higher and one of lower activity, see solid lines in left panels) denote a bistability that conditions the basic operation of working memory. The distance between the working point (*p*_EIN_ = *p*_Py_ = 0) and the saddle-node bifurcation (lower fold) reflects the intensity threshold (green-gray border in right panels in Figs. 2b, 3b) that separates transfer and memory behavior from nonresponsive behavior. For both stimulation targets a subcritical Hopf bifurcation give rise to a separatrix that bounds the basin of attraction of the upper (memory) state. Only if the system state is within that basin at the time the input signal is switched off, the stimulus will be memorized. This way, stimulation signals will be selected according to their temporal consistency and duration. Together with the intensity threshold this filter mechanism conditions the signal flow gating operation of the mCMC (see Kunze et al. 2017) for further information on the mechanisms underlying the basic operations of the canonical microcircuit model). Moreover, two stable limit cycles introduce sustained oscillations for stimulation of the Py (left panel in Fig. 3b), in contrast to the stimulation of EIN (left panel in Fig. 2b). These large-amplitude oscillations prevent the system from settling down within the separatrix when the stimulation ends (*p*_Py_ = 0). This explains the few sparse traces of the memory response behavior in the characteristic fingerprint (right panel in Fig. 3b). For further information on the system’s bifurcation structure, see Kunze et al. 2017 (stimulation of EIN) and Spiegler et al. 2010 (stimulation of Py).

In summary, the response behaviors indicate distinguishable operational roles for the separate input channels: While both channels gate information via an intensity threshold, only the EIN input channel, supposed to receive forward input, allows storing information. This differentiated processing documents the model’s capability to react differently to afferent information streams, as required in hierarchical setups.

### Concomitant stimulation modulates response behavior

Hierarchically interacting mCMCs might simultaneously receive feedforward and feedback information, effectively integrating low-level sensory and high-level conceptual information. Accordingly, we examined the transient feedforward stimulation of the EIN while concomitantly applying a constant feedback input, reflecting long holding times due to slower top-level processes (Bastos et al. 2015), to the Py of the mCMC model (see Fig. 2a).

We documented the consequently changed stimulation-induced response behaviors through bifurcation diagrams and characteristic fingerprints (Fig. 2). For increasing levels of constant feedback input to Py and transient forward input to the EIN, the range of memorized stimuli (orange area in Fig. 2b–f) increases. Meanwhile, the range of stimuli evoking nonresponsive behavior (green area in Fig. 2b–f) decreases, due to the lower stimulation intensity threshold (that is the upper edge of the green area). Hence, input to the Py causes formerly unresponsive stimuli to be perceived or even memorized (see markers in Fig. 2)—a behavior confirming the modulatory character of feedback information. These changes in transfer behavior are caused by a modified state space: According to the bifurcation diagrams (left plots in Fig. 2b–f), an increasing concomitant Py input shifts the lower fold bifurcation to smaller values (from *p*_EIN_ = 78 s^−1^ to *p*_EIN_ = 48 s^−1^) and widens the unstable Hopf cycle (i.e., a separatrix). This shorter distance between the working point (*p*_EIN_ = 0) and the lower fold bifurcation, determining the stimulation intensity threshold, and the larger space enclosed by the separatrix, effectively reducing the necessary stimulation duration to settle within the basin of attraction of the upper fixed point, promote the memory response behavior.

For comparison, we also considered the less plausible opposite case and applied increasing levels of constant input to EIN simultaneously to a transient stimulation of the Py. We find a modulatory effect that favors a stripe-like establishment of memory response behavior that is very sensitive to variations in stimulation duration (see Fig. 3b–e). Hence, there exists an asymmetry in the mutual modulatory influence of the input channels. The underlying bifurcation structure in the state space further clarifies this asymmetry and reveals configurations of constant input for which bistable behavior, a necessary condition for the memory behavior, is in fact present (see Fig. 3a).

In summary, simultaneous stimulation of the mCMC model with forward and feedback information causes an asymmetric mutual modulation of the response behaviors, which enriches the dynamic repertoire. Feedback input to the Py effectively modulates the system’s sensitivity to the driving feedforward input applied to the EIN.

Importantly, this modulatory effect causes different response behavior for identical stimuli and designates the feedback input as a *facilitation signal*. This facilitative feedback signal can be used in two ways to modify the stimulation-induced response behavior: (i) a non-perceivable stimulus (compare triangles in Fig. 2b, f) becomes perceivable and (ii) an either non-perceivable (circle) or non-memorizable stimulus (cross) becomes memorizable (Fig. 2b, f).

### Prototypical meta-circuits

So far we have shown how the minimal canonical circuit model processes feedforward and feedback information flows differently, and that feedback input can effectively regulate the access to basic operations for feedforward input. In the following, we derived prototypical meta-circuits of two interacting mCMCs that effectively make use of these processing traits in order to support priming and structure building.

**Fig. 4:**
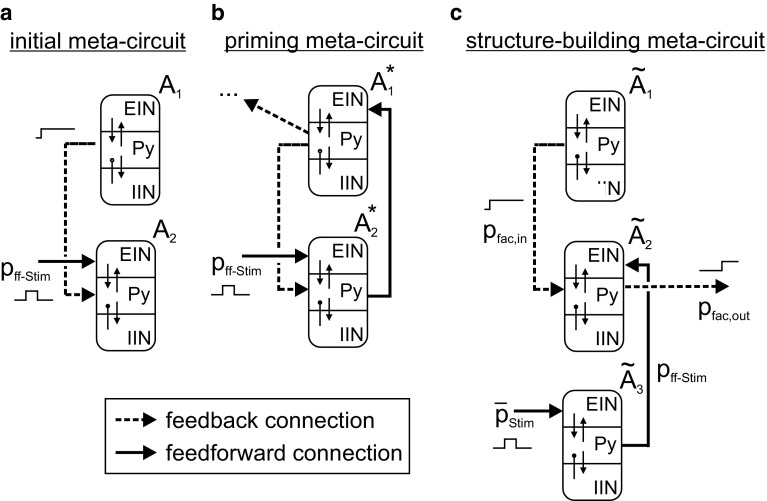
Prototypical meta-circuits of cooperating mCMCs. **a** In the initial meta-circuit a lower mCMC *A*_2_ receives a facilitative feedback signal (dashed line) from a higher mCMC A_1_ that modulates *A*_2_’s response to a feedforward stimulation *p*_ff-Stim_. Two computationally relevant derivations are investigated: **b** Facilitation modifies perception (priming), where a recurrent feedforward coupling from $$ A_{2}^{*} $$ to $$ A_{1}^{*} $$ (solid line) allows $$ A_{2}^{*} $$ to activate $$ A_{1}^{*} $$. The consequent facilitative feedback signal lowers the perceptual threshold in $$ A_{2}^{*} $$. Note that the facilitative feedback signal, conveyed by $$ A_{1}^{*} , $$ may also modify the perceptual threshold of yet another circuit. **c** Facilitation modifies memorization (structure building), where $$ \tilde{A}_{2} $$ is considered a higher microcircuit, but still receives a facilitative feedback signal, *p*_fac,in_, from $$ \tilde{A}_{1} $$. The feedback signal conditions the memorization of a feedforward stimulation *p*_ff-Stim_ arriving from a lower mCMC $$ \tilde{A}_{3} $$. In case of such an activation of $$ \tilde{A}_{2} $$, a facilitative feedback signal *p*_fac,out_ is conveyed to connected circuits, effectively cascading this local operation and supporting the incremental build-up of sustained activity patterns

In the initial meta-circuit (Fig. 4a), a higher level mCMC A_1_ conveys facilitative feedback signals to a lower-level mCMC A_2_, which also receives a feedforward stimulation *p*_ff-Stim_. The feedback signal is weak if A_1_ resides in a low activity state and high if *A*_1_ resides in a high activity state. This initial meta-circuit is the minimal canonical architecture that establishes a generic mechanism for state-dependent processing where the activity of one mCMC (here *A*_1_) governs the stimulation-induced response of another mCMC (here *A*_2_) with self-evident consequences in perception and memory. Notably, this state-dependent processing operation forms a potential building block for predictive coding by integrating conceptual model information (feedback input) with novel sensory information (feedforward input).

From this initial meta-circuit, we derived a prototypical meta-circuit for priming by introducing a feedforward connection from the lower to the higher mCMC (Fig. 4b). The inherent mechanism, adaptively shifting the perceptual threshold, is extensively studied for its boundary conditions in Sect. 3.4.

To derive a prototypical meta-circuit for structure building (Fig. 4c), we conceptually treat $\tilde{A}_{2}$ now as a higher level microcircuit that still receives a facilitative feedback signal *p*_fac,in_ from $\tilde{A}_{1}$. The feedforward stimulation signal *p*_ff-Stim_ is supposed to arrive from another lower-level mCMC $\tilde{A}_{3}$ which in turn receives stimulations to its EIN. The facilitative feedback signal *p*_fac,in_ will effectively regulate the availability of working memory in $\tilde{A}_{2}$, a mechanism studied in Sect. 3.5. By providing a facilitative signal *p*_fac,out_ to further connected circuits, this structure-building meta-circuit supports the establishment of spatiotemporal activation patterns and is used as a module for the syntax-parsing network examined in Sect. 3.6. Note that in real cortical circuitry, there will likely be also feedback connections from $\tilde{A}_{2}$ to $\tilde{A}_{3}$ that influence the transfer and memorization behavior of the latter and affect *p*_ff-Stim_. This may give rise to multiple hierarchical levels of structure building.

### Priming meta-circuit: dynamic shift of a perceptual threshold

**Fig. 5:**
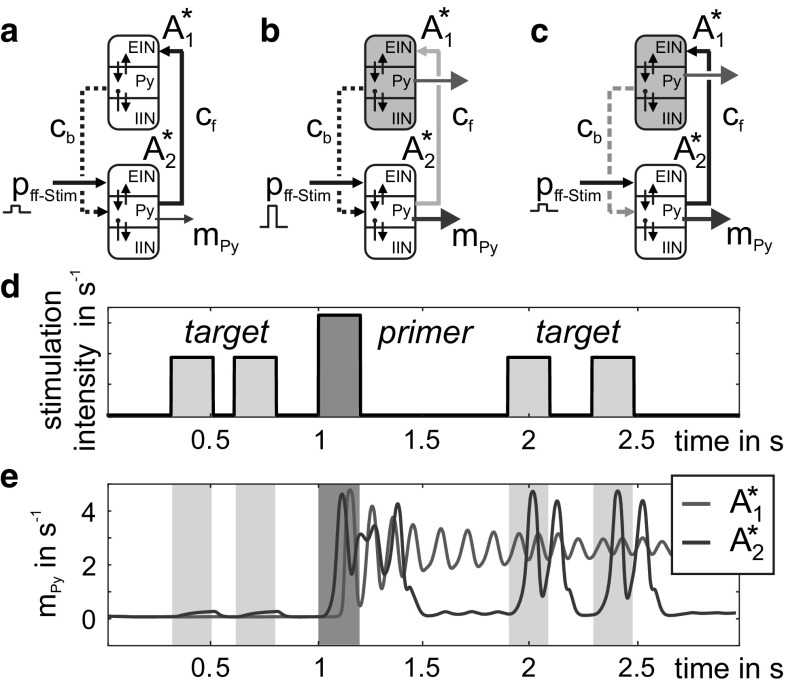
Principle mechanism for the dynamic shift of a perceptual threshold. **a** A target stimulus, applied to a mCMC $A_{2}^{*}$, is not able to cause a considerable output in $A_{2}^{*}$. However, as soon as a priming stimulus activates the higher mCMC $A_{1}^{*}$ (light blue line in **b**), $A_{1}^{*}$ emits a facilitative feedback signal (green dashed line in **c**) that allows $A_{2}^{*}$ to perceive the target stimulus. **d** Time course of afferent feedforward stimulations of the mCMC $A_{2}^{*}$. **e** Efferent signals of the mCMCs (color figure online)

Priming is an ubiquitous aspect of the brain’s processing abilities where one stimulus can influence the processing of subsequent stimulations (Kristjansson 2008). In the following, we examine a neural mechanism for priming that is based on the local cooperation of two mCMCs $A_{1}^{*}$ and $A_{2}^{*}$ (see Fig. 5a–c). Initially (Fig. 5a), an afferent *target* stimulus (light gray bar in Fig. 5d) does not considerably affect the output of a mCMC $A_{2}^{*}$, see Fig. 5e. However, a *priming* stimulus (dark gray bar in Fig. 5d) with higher intensity (and/or duration) causes a transient output in $A_{2}^{*}$ that activates the hierarchically higher mCMC $A_{1}^{*}$ (Fig. 5b). Through a feedback connection, the sustained high activation of $A_{1}^{*}$ now modulates the sensory sensitivity in $A_{2}^{*}$ and effectively shifts the perceptual threshold (Fig. 5c). Consequently, the same target stimuli that had no effect before priming now causes a considerable output in $A_{2}^{*}$ that is available for further processing. A deactivation of the higher level microcircuit $A_{1}^{*}$ would make $A_{2}^{*}$ insensitive to the target stimuli again. This homeostasis mechanism is readily implementable into the present model.

Compared to a single mCMC, the prototypical meta-circuit conceptually separates the basic operations of signal flow gating and working memory. The lower-level circuit $A_{2}^{*}$ primarily perceives and transmits salient inputs for further processing. Meanwhile, the higher level circuit $A_{1}^{*}$ modulates the processing in $A_{2}^{*}$ via facilitation that rests on $A_{1}^{*}$’s current activation state reflecting past processing events. In general, the feedback facilitation signal emitted by $A_{1}^{*}$ does not need to facilitate the same circuit that received the primer, i.e., self-priming, but may also facilitate processing in a different target-receiving microcircuit, representing the perception of another modality (as indicated in Fig. 4b).

We characterized the conditions of existence of this priming mechanism by closer inspection of a few key parameters.

- *The inter-circuit connectivity gains c*_*f*_*and c*_*b*_ The strength of forward and backward connections scale the signal strength at the targeted microcircuits. Forward connections regulate the switchover point of the dynamic threshold shift in the higher circuit $A_{1}^{*}$, that is when $A_{1}^{*}$ switches from a low to a high activation state. Backward connections scale the facilitative feedback signal that modulates the response behavior in the lower circuit $A_{2}^{*}$.
- *The afferent stimuli* Intensity and duration of the target stimulus decide whether a stimulus can be perceived or not. The priming stimulus, being salient in terms of its intensity (or duration), evokes a sustained high activation (i.e., memory behavior) in $A_{1}^{*}$, causing the facilitative feedback signal. We examine the relationship between target and priming stimuli in terms of varying intensity while keeping their durations equal.
- *Individual adaptation of the microcircuits* In an earlier study we showed that individual levels of the local network balance can bias the response dynamics of mCMCs (Kunze et al. 2017). In particular, a slight increase of inhibition, relative to the default parameterization, favors the transfer behavior, while a slight decrease of inhibition favors the memory behavior. Accordingly, we examined how inhibitory synaptic gains H_i_, characterizing each population’s inhibitory synaptic response, constrained the working range of the priming mechanism.

**Fig. 6:**
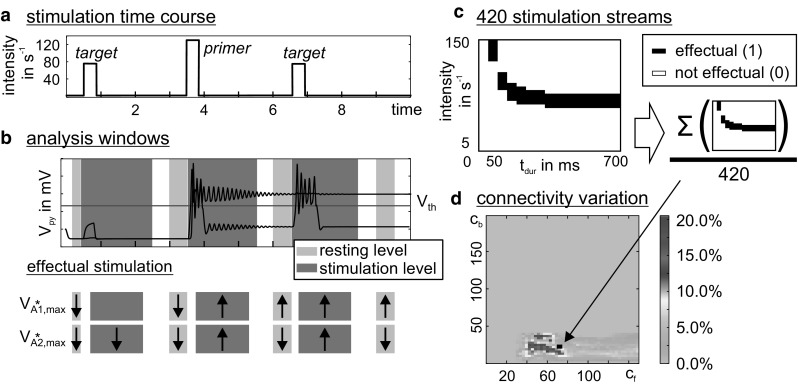
Analysis of the priming mechanism. **a** For the characterization of the priming mechanism, we applied stimulation streams that comprised two target stimuli separated by a priming stimulus. **b** We evaluated the response behaviors of $A_{1}^{*}$ and $A_{2}^{*}$ by comparing *V*_Py_ to a firing threshold of 4 mV in seven analysis windows: before, after (i.e., resting level, light gray sections), and during a stimulation (dark gray sections). As indicated by the arrows, a stimulation stream was said to be effectual, i.e., evoked a perceptual threshold shift, if (a) the priming evoked a sustained high activation in the higher mCMC $A_{1}^{*}$, but not $A_{2}^{*}$, and (b) the target stimulus evoked a transient high activation only after priming. **c** Target stimuli of different duration (*t*_dur_) and intensity were applied. The sum of all effectual stimulation streams was scaled by the total number of considered stimulation streams. **d** This color-coded percentage was mapped to a range of connectivity gains *c*_f_ and *c*_b_ in order to characterize their constraining influence on the priming mechanism

**Fig. 7:**
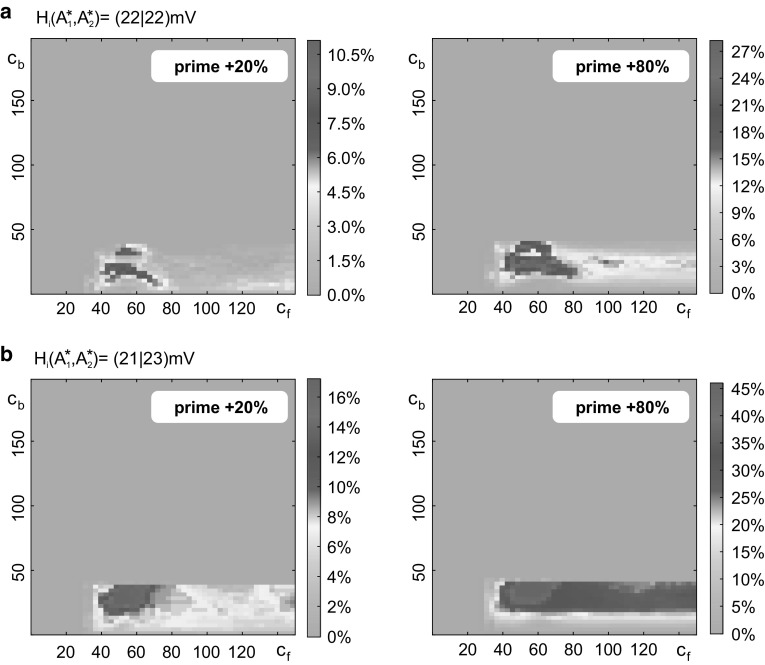
Impact of connectivity gains, priming intensity and network balance to the priming mechanism. Connectivity gains constrain effectual stimulation streams. Each plot varies over the connectivity gains *c*_f_ and *c*_b_ and sums over stimulations of different length and intensity (see Fig. 6). Colors code the percentage of effectual stimulation streams that evoked priming. Priming and target stimulus were equally long, but the primer’s intensity was 20% (left column) or 80% (right column) larger. Larger primers lead to more effectual stimulations streams both in terms of the maximum rate and the range of suited connectivity gains. Compared to the default configuration of inhibitory synaptic gains (**a**), a slight decrease of inhibition in $A_{1}^{*}$ combined with an increase of inhibition in $A_{2}^{*}$ (**b**) enhances the maximum rate of effectual stimulation streams and the range of suited connectivity gains. Note the different color scaling in the single subplots and that the percentages are specific for the chosen stimulation parameter ranges (see Table 2)

To evaluate these critical parameters in the priming mechanism, we applied individual stimulation streams, each composed of two target stimuli separated by a priming stimulus (see Fig. 6a), to the lower-level circuit $A_{2}^{*}$ of the priming meta-circuit. The response behaviors of $A_{1}^{*}$ and $A_{2}^{*}$ were assessed in seven analysis windows (before, during, and after the individual stimuli, see Fig. 6b) by comparing the membrane potential of the pyramidal cells with a firing threshold. A stimulation stream was defined as *effectual* (i.e., evoking a shift of the perceptual threshold), if (a) the priming stimulus evoked a sustained high activation in $A_{1}^{*}$, but not in $A_{2}^{*}$ and (b) the target stimulus evoked a supra-threshold transient deflection in $A_{2}^{*}$ only after, but not before, the priming stimulus (see Fig. 6b). Whereas the target stimuli varied in intensity and duration, the priming stimulus was of equal length but of higher intensity (see Table 2) in relation to the target stimuli. Supraliminal target stimuli that evoked supra-threshold deflections (i.e., transfer or memory behavior) in $A_{2}^{*}$ without priming were disregarded. For all other stimulation streams we mapped the percentage of effectual stimulation streams (see Fig. 6c) to the inter-circuit connectivity gains *c*_f_ and *c*_b_, see Fig. 7. This stimulation procedure was repeated with modified inhibitory synaptic gains *H*_i_ in $A_{1}^{*}$ and $A_{2}^{*}$, signifying an altered network balance ratio compared to the default network balance (Jansen and Rit 1995). Table 2 lists the varied parameter values.

**Table 2:** Parameter values for the assessment of the perceptual priming mechanism

Parameter		
	Target stimulus	Priming stimulus
*Stimulus duration*		
Minimum	50 ms	50 ms
Step	50 ms	50 ms
Maximum	700 ms	700 ms
*Stimulus intensity*		
Minimum	5 s^−1^	Target + (20, 80) %
Step	5 s^−1^	5 s^−1^
Maximum	150 s^−1^	Target + (20, 80) %

	*c*_f_	*c*_b_
*Connectivity gains*		
Minimum	0	0
Step	3	3
Maximum	150	200

Inhibitory synaptic gain *H*_i_	Default (Fig. 7a)	$A_{1}^{*}$ and $A_{2}^{*}$ bias (Fig. 7b)
*H*_i_ ($A_{1}^{*}$) (mV)	22	21
*H*_i_ ($A_{2}^{*}$) (mV)	22	23

**Fig. 8:**
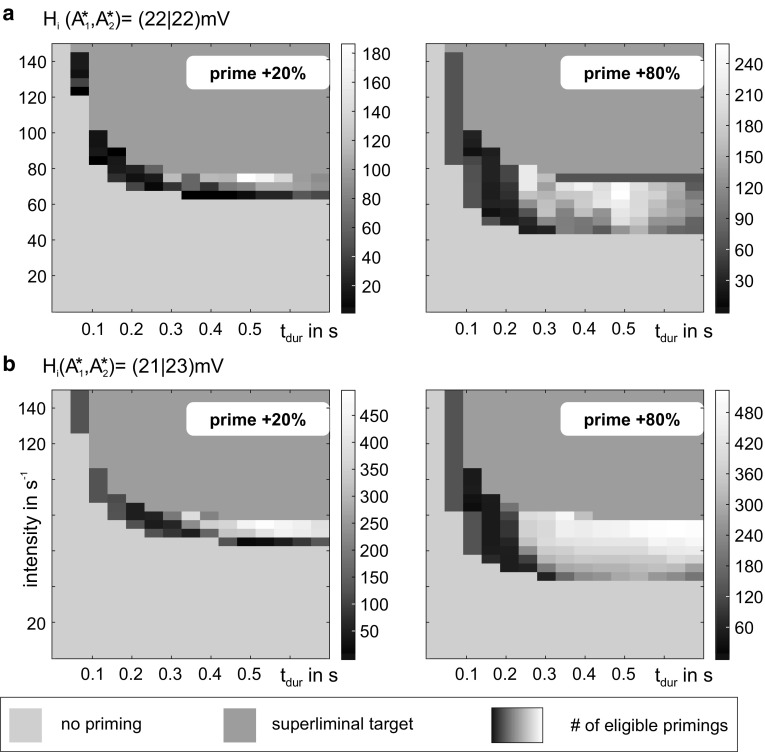
Characterization of stimuli that evoked a perceptual threshold shift. Each subplot varies over stimulus duration and intensity and shows the number of effectual stimulations (summed above all *c*_f_–*c*_b_ combinations) in grayscales. Supraliminal target stimuli, i.e., very strong or long stimuli, were already recognized, making priming superfluous (sandy coloring). Subliminal target stimuli, i.e., weak and brief target stimuli, were not suited to initiate the priming, because they failed to evoke a response memory behavior in the higher circuit $A_{1}^{*}$ (light blue coloring). Columns relate to the priming intensity, where the priming stimulus is 20% (80%) larger than the target stimulus. A stronger priming intensity increased the range of perceived target stimuli. Compared to the default configuration of the inhibitory synaptic gains (**a**), a decreased inhibitory synaptic gain in the higher circuit $A_{1}^{*}$ combined with an increased inhibitory synaptic gain in the lower circuit $A_{2}^{*}$ did not only promote the number of perceived target stimuli, but also their invariance to variations in intensity or duration, marked by the larger range of effectual stimulations. Note the different grayscales for each subplot

**Fig. 9:**
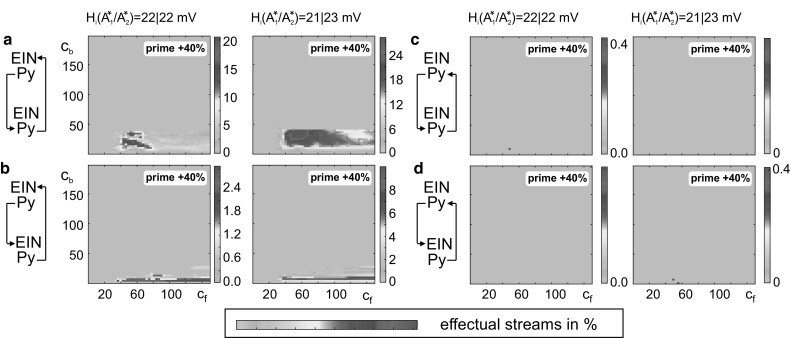
Importance of the prototypical meta-circuits’ topology. **a** For the priming mechanism, we considered a specific topology within the prototypical meta-circuit, where the higher microcircuit connected to the lower microcircuit via a backward connection, i.e., targeting the Py, and the lower microcircuit connects to the higher microcircuit via a forward connection, i.e., targeting the EIN. This arrangement supported a functional specialization: the forward connection allowed storing events and favors memory in the higher circuit. The backward connection allowed the lower microcircuit to perform a dynamic signal flow gating. In contrast, alternative topologies, such as consideration of pure feedforward (**b**), pure feedback connections (**c**), or a permutation of feed forward and feedback connections (**d**) failed to support the priming mechanism, but may be relevant for other cooperative neural operations

The connectivity gains *c*_f_ and *c*_b_ constrain the priming mechanism (see Fig. 7). Effectual stimulation streams occur for *c*_f_ > 30, signifying a minimum feedforward strength in order to convey $A_{2}^{*}$’s activation to the higher level circuit during priming, and for *c*_b_ < 45, signifying a maximum feedback strength in order to limit the facilitation in the lower-level circuit and keep it sensitive to further stimulation (i.e., prevent self-activation). Note, however, that the exact values are subject to the chosen model parameterization. These connectivity constraints were observed for a varying intensity of the priming stimulus (20% and 80%, respectively) and for all considered configurations of the inhibitory synaptic gains *H*_i_ (Fig. 7). The percentage of the effectual stimulation streams increased with the relative strength of priming and target stimulus for all considered configurations of the inhibitory synaptic gains H_i_. For the default configuration (Fig. 7a), high rates of effectual stimulation streams were restricted to a small range of connectivity values. A slight decrease in inhibition in the higher circuit $A_{1}^{*}$ and simultaneous increase in inhibition in the lower circuit $A_{2}^{*}$ maximized the range of suited connectivity values and the rate of effectual stimulation streams (Fig. 7b). We further identified those target stimuli that evoked the perceptual threshold shift that is characteristic for the priming mechanism (see Fig. 8). Strong and long target stimuli (i.e., supraliminal) were already recognized, making priming superfluous (sandy coloring). Furthermore, weak and brief target stimuli were not suited to initiate the priming, as they fail to evoke a memory behavior in the higher level circuit $A_{1}^{*}$ (light blue coloring).Tuning the inhibitory synaptic gains increased the range of suited stimulation parameters. Importantly, the described priming mechanism is not achievable in alternative topologies of the local network (see Fig. 9).

In summary, we propose a mechanism for priming in a prototypical meta-circuit of two mCMCs that is based on the effect of top-down facilitation. The mechanism rests on the constructive cooperation of the involved mCMCs and predicts their conceptual specialization to either *signal gating*, thereby avoiding insensitivity to future stimuli, or *memorization*, to guide further information processing through facilitation. This functional specialization can be biased by means of the local ratio of excitation and inhibition in the involved microcircuits. The topology of feedforward and feedback connections within the meta-circuit and the salience of stimuli constrain the priming mechanism.

### Structure-building meta-circuit

In the previous section we showed how top-down facilitation via feedback connections permits the dynamic perception of subliminal stimuli. In the following, we show how a facilitation signal conditions the memorization of stimulation events in the structure-building meta-circuit (Fig. 4c).

**Fig. 10:**
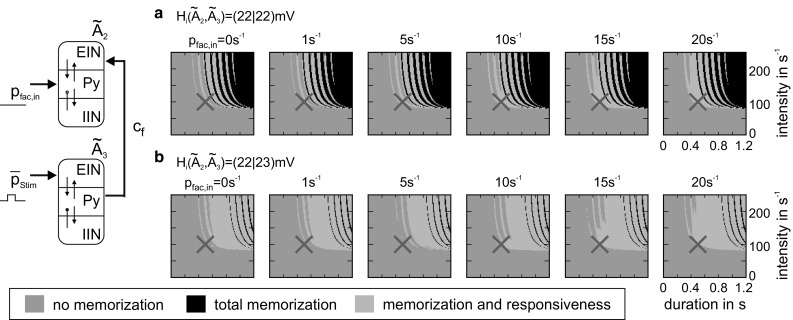
Facilitative signal enable the memorization of stimulation events. A mCMC $\tilde{A}_{3}$ received stimuli of distinct length and intensity and in turn stimulated a mCMC $\tilde{A}_{2}$ via a forward connection. **a** For default inhibitory synaptic gains, few stimuli to $\tilde{A}_{3}$ enable the selective activation of $\tilde{A}_{2}$ while keeping $\tilde{A}_{3}$ responsive (i.e., *memorization and responsiveness*, green area). Weak and brief stimuli fail to activate $\tilde{A}_{2}$ (*no memorization*, gray area), whereas strong and long stimuli activate both $\tilde{A}_{2}$ and $\tilde{A}_{3}$ (*total memorization*, black area). Higher levels of the facilitative feedback signal *p*_fac,in_ promote the selective activation of $\tilde{A}_{2}$. **b** A slight increase of the inhibitory synaptic gain H_i_, favoring a transfer response behavior in $\tilde{A}_{3}$, promotes the selective activation of $\tilde{A}_{2}$. Red crosses denote an exemplary stimulation of defined intensity and duration applied to $\tilde{A}_{3}$ (color figure online)

In the meta-circuit for structure building, we applied transient rectangular feedforward stimuli to $\tilde{A}_{3}$ and mapped the consequent functional states of $\tilde{A}_{2}$ and $\tilde{A}_{3}$ for increasing levels of facilitative external feedback *p*_fac,in_ (see Fig. 10). For the default inhibitory synaptic gains, weak and short stimuli are not able to activate $\tilde{A}_{2}$ (*no memorization*, gray area) and strong and long stimuli activate both $\tilde{A}_{2}$ and $\tilde{A}_{3}$ (*total memorization*, black area). Few stimuli are able to selectively activate $\tilde{A}_{2}$, but avoid a sustained high activity of $\tilde{A}_{3}$ and thus preserve its responsiveness to further input (*memorization and responsiveness*, green area). Higher levels of the facilitative feedback signal *p*_fac,in_ promote the selective activation of $\tilde{A}_{2}$ (red crosses in Fig. 10a). A slight increase of inhibition in $\tilde{A}_{3}$ further promotes this selective activation (see Fig. 10b), effectively favoring a transfer response behavior (Kunze et al. 2017). In summary, the top-down facilitation signal *p*_fac,in_ conditions the establishment of sustained activity in one part (i.e., $\tilde{A}_{2}$) of the prototypical meta-circuit for structure building, while preserving its input sensitivity (i.e., $\tilde{A}_{3}$). In the following, we exemplify how this local operation supports the sequential and selective establishment of spatiotemporal structures in a simple realization of incremental (i.e., word by word) syntax parsing during the perception of a sentence.

### Syntax-parsing language network

Understanding a spoken or written sentence comprises many processing steps, including acoustic perception, mapping of syntactic and semantic information, and inclusion of additional information (i.e., context and individual experience) (Friederici 2002).This process of sentence perception manifests itself as the sequential activation of neural populations in the involved neocortical areas (Kunze et al. 2017; Rolls and Deco 2015; Pulvermüller et al. 2014). In the following example, we revisit a language model (Kunze et al. 2017) which focuses on the representation of syntactic information in a distributed network of mCMCs. In particular, it accounts for the decoding and temporal storage of syntactic information, here referred to as *parsing*, that is necessary for further processing (Friederici 2002). Words are thought to be represented by word webs, comprising numerous nodes for all aspects of the word, including word forms, various semantic associations, and one or several syntactic roles. Here, we adopt a simplified model containing, for each word, only nodes for the possible syntactic roles (i.e., subject, verb, and object) that follow the principle of place coding (Rolls and Deco 2015), and one node for the rest of the word web. Each node is represented by a mCMC.

Consecutive afferent word information, stemming from auditory areas of the cerebral cortex, incrementally evokes a sustained higher activation in the respective word-representing microcircuits. This generic structure-building mechanism, referred to as *dynamic recruitment*, emphasizes the potential freedom of the parse concerning the number and order of its elements (Kunze et al. 2017). The analysis of this initial model revealed the following issues:

- *Multiple instantiations of single words* Due to a single object-module, comprising all known nouns of an individual’s vocabulary, the initial model was not able to represent a repeated instantiation of the same word, as in the sentence “I draw a wall on a wall”. A recognized word activated the respective microcircuit, which became insensitive to further stimulation—and was hence not available for further recruitment.
- *Simple syntax structure* The modularized organization of the network constrained more complex syntax structures. The selection and flexibility of order of syntactic categories required the repetition of entire word-grouping modules, implying a redundancy of words and the effort for their maintenance.
- *Self-activation by means of pre-activity* Due to the linear superposition of pre-activation, transmitted by connected microcircuits, and afferent word information in a single population of the mCMC, an aggregation of pre-activation allowed a self-activation of a mCMC.

**Fig. 11:**
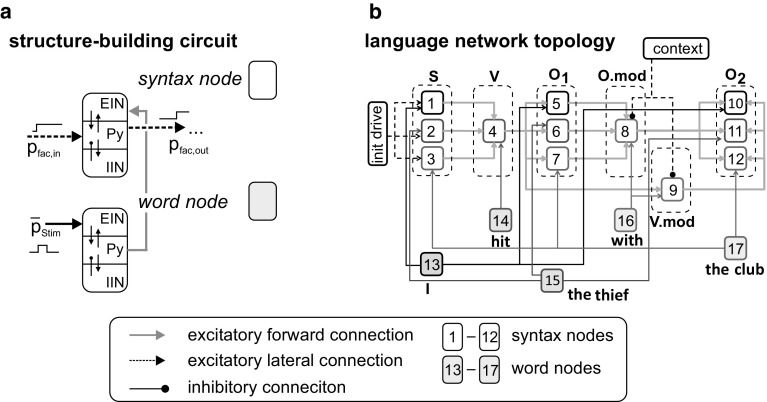
Architecture of the syntax-parsing network. **a** The structure-building meta-circuit was used as a building block for the syntax-parsing network. We interpreted the higher mCMC as a *syntax node*, representing syntactic categories, and the lower as a *word node*, representing single words of a vocabulary. Note that there can be several syntax nodes for each role the word can assume. **b** In the syntax-parsing network 17 interacting mCMCs represented either syntax nodes (1–12) or word nodes (13–17). One word and several syntax nodes form a word web (color-coded), while all syntax node of the same kind form a syntactic pool (dotted frames). Word nodes project unidirectionally to syntax nodes via feedforward connections (black arrows). Syntax nodes in different pools are interconnected by lateral connections (gray arrows) and exchange facilitative signals that condition the establishment of sustained activity patterns. Mutual inhibition within syntax pools ensures that a particular syntactic role is only assumed by one word (not shown for simplicity). Contextual information assists the semantic interpretation (dashed arrows)

In the following, we advance this model and capitalize our findings on state-dependent processing and functional specialization of mCMCs in hierarchical networks. We designed a distributed syntax-parsing network with 17 interacting mCMCs as network nodes (see Fig. 11b)—effectively implementing structure-building meta-circuits (see Fig. 11a). Each word web comprises one *word node* (nodes 13–17 in Fig. 11b) representing the acoustic word form and between 1 and 3 *syntax nodes* (nodes 1–12 in Fig. 11b) for the potential syntactic roles the word can assume. For instance, the word web of the noun “thief” contains subject and object nodes (see Fig. 11b). Other constituents of the word webs, like the numerous semantic associations, are omitted for simplicity. Within each word web, the output of the word node (Py) projects to the inputs (EIN) of the syntax nodes. Backward connections from the syntax nodes to the rest of the word webs (including the word nodes), which are necessary to remap syntactic roles to the actual semantic content, are omitted for simplicity. All syntax nodes representing a particular syntactic role (e.g., subject) in the different word webs form *syntax pools*, where they are connected by mutually inhibitory connections (omitted in Fig. 11b for the sake of clarity). This ensures that a particular role can only be assumed by one word (while a word can fulfill several roles, like in the sentence “I draw a wall on a wall”). Finally, the syntax pools are connected according to the syntactic rules (e.g., subjects to verbs, but not to objects). If two syntax pools are connected, all syntax nodes in the source pool connect in an all-to-all fashion to the Py populations of all syntax nodes in the target pool. The projection to the Py causes an increase of excitability (facilitation) without actually activating the node (thus establishing expectation). Similar to the model of Kunze et al. (2017), contextual information guides structure building by means of inhibitory connections in order to address ambiguity in sentences.

For the simulation of sentence perception, the word nodes perceived brief acoustic inputs and transmitted their transient activation to the respective syntax nodes. Among the syntax nodes only those that had been previously syntactically predicted, i.e., received a lateral facilitative signal, became activated. The initial expectation to perceive a sentence is modeled as an initial driving signal received by the subject-reflecting syntax node. Important parameters of the language model are summarized in Table 3.

**Table 3:** Important parameters of the language model

Connections from word to syntax nodes (excitatory Py → EIN)	*c*_f_	34…54	Contextual information	*p*_inh_	3.8 s^−1^
Connections between syntax nodes of different pools (excitatory Py → Py)	*c*_lat_	5	Intensity of acoustic input	*p*_stim_	140 s^−1^
Connections between syntax nodes within pools (inhibitory Py → IIN)	*c*_inh_	10	Duration of acoustic input	*t*_dur_	500 ms

**Fig. 12:**
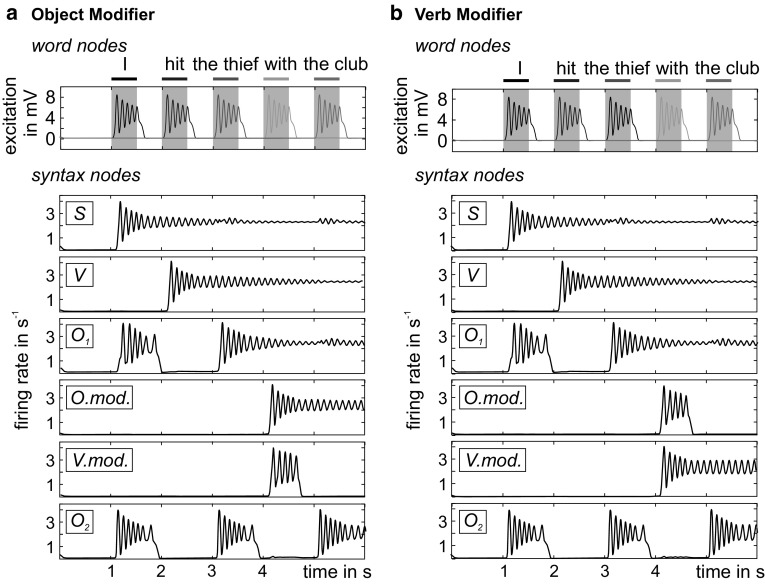
Analysis of the syntax-parsing network. **a** Top plot: word nodes respond to their consecutive stimulation (gray areas) and selectively activate syntax nodes. Bottom plot: activations of syntactic pools as sums of their respective syntax nodes. **b** Changing contextual information, i.e., blocking the object modifier, changes the interpretation of the sentence

For the simulated parsing process, we considered the sentence “I hit the thief with the club” (Kunze et al. 2017). During the parsing process, word nodes responded to their consecutive stimulation (top plot in Fig. 12a) and selectively activated the syntax nodes (bottom plots in Fig. 12a)—providing a sustained activity trace at the end of the parsing process. This way, information is stored about the activated syntactic roles (cumulative signals of each syntactic pool) and the actual word web linked to that role (which node within a syntactic pool is activated, note that due to mutual inhibition only one node per pool can be active).

Depending on the contextual information, the phrase *with the club* further specified the verb *hit*, i.e., operating as an adverbial phrase, or the object *thief*, i.e., operating as an adjective phrase (Kunze et al. 2017). Parsing the sentence “the thief hit the thief with the club” is also possible, due to the separation of lexical word information and syntactic categories that allows multiple instantiations of words independent of their syntactic categories.

The feature of the structure-building meta-circuit considerably increases the face validity of the syntax-parsing network as it allows more complex syntactic structures to be considered. The perception of single words (e.g., thief) is transient in the word nodes and excites all connected syntax nodes (e.g., S, O_1_, O_2_). However, the excitation only remains for syntactically predicted syntax nodes (S), but vanishes for unpredicted nodes that do not receive a facilitative signal (O_1_, O_2_).

## Discussion

Contributing to the concept of the minimal canonical microcircuit (mCMC), we showed that afferent feedforward and feedback stimulations led to different responses. While both streams are gated by an intensity threshold, only feedforward input, potentially representing novel sensory information, can be stored for prospective processing. Moreover, constant modulatory feedback input, reflecting longer holding times due to slower top-level processes (Bastos et al. 2015), modifies the system’s sensitivity to simultaneously applied driving feedforward input, effectively conditioning the access to basic operations. This observation is in line with empirical studies showing the modulatory effect of top-down information (Spillmann et al. 2015). The facilitative feedback signal is thus a flexible and effective way to regulate information processing without the need for synaptic adaptations. We find that both feedforward and feedback input can modulate the respective other, though in an asymmetric way, supporting the notion that feedforward information is not strictly driving and feedback information is not strictly modulatory (Covic and Sherman 2011; Bastos et al. 2015).

On the basis of cooperating mCMCs we identified state-dependent processing operations that are similar to the concept of conditioned associations in cell assembly theory (Wennekers et al. 2006), further proving the compatibility of basic operations in mCMCs and cell assembly theory (Palm et al. 2014; Pulvermüller et al. 2014). Here, state-dependent processing, mediated by a facilitative feedback signal, rests on the basic operation of working memory that allows one mCMC to store processing events over time and keep this information available for future processing steps. The resulting temporal processing history gives rise to a broad set of conceivable adaptive processing mechanisms, two of which we further exemplified in this paper. Note that here we consider persistent neural activity as basis for working memory, which, alternatively or additionally, may also rest on potentiated synapses.

The initial prototypical meta-circuit (Fig. 4a) is the minimal model for this form of state-dependent processing as it conceptually separates the basic operations of working memory and signal flow gating and assigns them to separate mCMCs, which functionally specialize to distinct basic operation. In a recent study, we showed that the inhibitory synaptic gain biases the response behavior in a canonical microcircuit model (Kunze et al. 2017). In the present study, we showed how the adaptation of inhibitory synaptic gains favored the functional specialization of areas and that this adaptation improves the reliability and efficiency of the facilitative feedback signal. Because this functional specialization does not require any structural changes, the possibility to dynamically re-assign the dominant basic operations of mCMCs also underscores the task-based adaptability of networks of mCMCs and eventually the cortical matter. This might be an important aspect if a part of the cortex needs to adapt to a new functionality, such as during remapping after stroke (Cheng et al. 2016) or acquisition of new skills. Our model suggests that functional specialization is reversible on different time scales and through different modalities that affect the local ratio of excitation and inhibition, referred to as local network balance. Although we chose inhibitory synaptic gains, lumping together parameters of synaptic transmission (Jansen and Rit 1995), other neurobiologically plausible means to regulate the local network balance include the received neural activity, synaptic plasticity, pharmacological neuromodulation, and electrical brain stimulation (Kunze et al. 2016). While the neural causes that give rise to the behavioral effects of electrical brain stimulation are still unclear (Fertonani and Miniussi 2016), one explanation might be that anodal tDCS increases the excitability of neural population by shifting the network balance. Our findings on the consequences of a shifted network balance are thus an interesting starting point for the investigation of the functional mechanisms of electrical brain stimulation.

Our study demonstrates the value of state-dependent processing through facilitative feedback signals in two applications, namely priming and structure building. Priming refers to a behavioral phenomenon, generally stating that past experience modifies the current processing performance (Tulving and Schacter 1990; Schacter and Buckner 1998; Kristjansson 2008). Because of its ubiquity, and despite its behavioral diversity, priming may depend on a small set of generic neural mechanisms that are individualized for the respective task. Based on the dependence on past states, priming must involve some kind of storage and was proposed as a special form of memory (Tulving and Schacter 1990). Here, we proposed a prototypical meta-circuit embodying a mechanism for perceptual priming that rests on the dynamic shift of a perceptual threshold by means of a facilitative feedback signal. Analysis of topology, connectivity weights, and stimulation characteristics suggested that the meta-circuit’s architecture must support a functional specialization to working memory and signal flow gating, i.e., feature a forward connection to evoke a memorized high activity and a feedback connection for facilitation. Notably, here we only considered repetition priming (or self-priming), where a single mCMC receives both target and priming stimuli. In reality, the ‘single-channel’ circuit treated here may be replaced by multiple channels, where the connection matrix from *A*_1_ to *A*_2_ (Fig. 5b) defines the association between priming and primed inputs. Note also that in addition (and in competition) to priming there will also be sensory adaptation (or habituation), causing attenuation of the processing of repeated stimuli. This process is likely to rely on short term synaptic plasticity, which can be easily incorporated into the mCMC model (see, e.g., Wang and Knösche 2013).

Importantly, the mechanism presented here is based solely on the sustained activation of the memory node(s) *A*_1_ of the primer representation, which automatically pre-activate the perception nodes *A*_2_ of the target representation. No further top-down influence from wider networks is considered. However, attention may influence and even counteract priming mechanisms (e.g., Keane et al. 2015). In our model, such top-down modulation could be realized by additional pre-excitation or inhibition on the perception or memory nodes. Moreover, priming may in turn also affect the attentional focus.

Interestingly, similar to our priming scenario, Ardid et al. (2007) proposed a model for attentional processing that investigates the hierarchical cooperation of the prefrontal cortex and the visual middle-temporal area. The similarity of both computational approaches supports a supposed common mechanism underlying top-down attention processing and perceptual priming (Kristjansson 2008). Both works describe a neural circuit for the modulation of perception that is composed of two different modules: one representing a memory system and one a sensory system. However, the canonicity of the circuit is described on different scales. Ardid considers the entire circuit as canonical, because it reflects diverse attentional effects. Here, we assume each single module to be canonical, as it reflects basic processing operations (Kunze et al. 2017). The meta-circuit of this study would therefore reflect the organizational level of Ardid’s neural circuit. Hence, whereas Ardid’s work enlightens numerous cognitive attentional effects, our work extends the mechanistic understanding of the intermodular interaction by even simpler basic operations.

We showed that the facilitative feedback signal conditions the memorization of stimulations. We then applied this finding in a syntax-parsing network that effectively cascades multiple structure-building meta-circuits. In the syntax-parsing network, word-representing mCMCs selectively activated higher-order mCMCs that represented syntactic categories. Both kinds of mCMCs are thought to be part of a word web (Pulvermüller 2002), which collectively represents features of a distinct object and is, for instance, excitable through the reception of acoustic word information. The word webs are activated by perceived word information transiently and independently from the expected syntactic category. This allows the word to be used multiple times in a sentence, extending a former syntax-parsing network (Kunze et al. 2017). In contrast, the sustained activation of syntax nodes, selectively excited by the word nodes, causes predictions among the syntax nodes that guide the parsing according to syntax rules. In principle, both afferent word information and syntax predictions are unspecific (e.g., the word *drink* can be an object or a verb and, as an object, can be succeeded by many syntactic categories). Nevertheless, through the integration of unspecific word information and unspecific syntactic predictions, the syntax-parsing network yields a specific syntax prototype. Potential ambiguities are resolved by contextual information. The separation of minimal redundant word nodes and syntactic nodes suggests the construction of detailed syntax structures up to the point of reflecting phrase structures and symbolic operations (Chomsky 1995); a point that needs to be further investigated. With these developments, the present syntax-parsing network signifies a promising step toward a mechanistic link between the research on canonical microcircuits and linguistics. Here, we only consider a single type of syntax prototype (i.e., subject–verb–object–object). It is flexible in its structure, though, by distinguishing adjective and adverbial phrases and the optional use of the second object. However, our model is only a little cutout of a much more complex network that allows greater variety of prototypes through higher numbers of syntax nodes and their connections.

In the literature, many other binding mechanisms have been proposed. For instance, Eliasmith et al. (2012) describe a neural network of single spiking neurons that, besides other cognitive tasks, provides for serial working memory. In their neural model of working memory, an item is explicitly bound to a specific position and can be recalled by rerouting this neural activity. In contrast, our model stores the syntactic roles of the items. This ensures the necessary flexibility for coding sentences. Although the proposed mechanisms may not yet reflect the entire complexity of psycholinguistics, we show how generic principles, such as binding, can be realized in models of neural interaction.

Common to both phenomena of priming and structure building is the notion that past processing steps modify the current processing. In particular, the facilitative feedback, arising from the sustained activation of one microcircuit, either shifts the perceptual threshold (perceptual priming) or permits the stimulation-induced sustained activation (structure building) in another microcircuit. In both examples, the facilitative feedback signals confine potential subsequent processing steps so that non-facilitated, that is, unpredicted stimulations are ineffective. This ability of anticipation, together with the notion that the mCMC integrates feedforward stimulations (i.e., sensory or prior information) with feedback information (i.e., conceptual or model information), suggests a conceptual proximity of state-dependent processing in mCMCs to predictive coding (Mumford 1992; Rao and Ballard 1999; Friston 2005; Shipp 2016). However, in contrast to other established canonical microcircuit models that embody predictive coding (Bastos et al. 2012, 2015), our framework does not consider the explicit exchange of prediction errors, potentially reflecting different mechanisms (Palm et al. 2014).

With this work, we follow the notion that the brain’s information processing rests on a hierarchically organized network structure across multiple organizational scales (Stam and van Straaten 2012). The concept of canonical microcircuits mostly refers to the level of cortical columns (Douglas and Martin 2004; Haeusler et al. 2009). However, the mCMC considered here emphasizes positive and negative feedback loops and can thus be regarded as valid on multiple spatial levels of brain networks, ranging from single neurons to entire cortical areas. Accordingly, we interpret our results as not specific for a distinct type of neocortex (Beul and Hilgetag 2014), or for elaborated architectures (Haeusler et al. 2009), but as part of a set of generic, though not trivial, neural mechanisms of the brain’s processing.

The mCMC and the derived meta-circuits presented here are designed to bear a minimum of structural features. They disregard most features of laminar organization, such as the distinction between supragranular (layers II/III) and subgranular (layers V/VI) cell populations. More detailed architectures have been proposed for, both, rate-based (Wang and Knösche 2013; Joglekar et al. 2018) and spiking neuron models (Lee et al. 2013). A very complex and realistic spiking neuron model for a local cortical microcircuit (80,000 neurons and 300 million synapses) incorporating a wealth of microanatomical knowledge taken from the literature was proposed by Potjans and Diesmann (2014). Models with realistic laminar organization provide the ground for investigating a wider range of phenomena. For example, the separation of subgranular and supragranular circuits and their respective tuning to alpha and gamma band oscillations allowed Mejias et al. (2016) to reproduce several experimental findings concerning the modulation and coupling of these oscillations. It remains to be investigated in future work to what extent the dynamics and, most importantly, the functional behavior of mCMCs as described in this work would be modified by such more detailed models.

Also, in this study, we focused on the modulatory effect of excitatory input channels, although long-range neural projections do target inhibitory interneurons (IIN) as well. While a thorough investigation of their influence on the effects is left to future studies, we can draw some preliminary conclusions from our present and previous studies (Kunze et al. 2017). Due to the mathematical formulation, a time-constant stimulation of the IIN within the linear section of the sigmoidal activation function is proportional to an increase in the inhibitory synaptic gain. Although this view disregards the effect of potential inter-circuit inhibitory feedback loops, it allows assessing the effect of an additional inhibitory stimulation. On the level of a single mCMC, an inhibitory stimulation would suppress memory behavior and favor transfer behavior (Kunze et al. 2017). In the priming meta-circuit canonical microcircuit, a stimulation would enhance priming effects, if it targets the lower-level mCMC *A*_2_* (Fig. 7), and in turn diminish priming effects if it targets the higher-level mCMC *A*_1_*.

## Conclusion

Our results support the notion of conceptual compatibility between mCMCs and operating cell assemblies. This leads to the idea that mCMCs may serve as biologically plausible nodes in models of hierarchically operating cell assemblies, effectively addressing the structural realization of cell assemblies in the neocortical matter. Such networks of canonical microcircuits—that have already been used for EEG, MEG, and fMRI replication—may support systematic experiment-based investigations of cognitive functions. Exemplifying this notion, we found mechanistic evidence that cognitive priming involves the dynamic shift of a perceptual threshold and that structure-building computations are likely subfunctions in a neural network for syntax parsing.

## Electronic supplementary material

Below is the link to the electronic supplementary material.

Supplementary material 1 (JPEG 42 kb)

Supplementary material 2 (JPEG 38 kb)

## Footnotes

1 Note that the term “interneuron” is often reserved for inhibitory cells. Here, an interneuron is any neuron that receives local input and projects to nearby cells. This nomenclature has been used by previous authors, including Jansen and Rit (1995).
