# Supplementary figures and images for: Emergence of cognitive priming and structure building from the hierarchical interaction of canonical microcircuit models
Source: Biol Cybern. 2019 Feb 14;113(3):273–91. doi: 10.1007/s00422-019-00792-y (PMC6510829; doi:10.1007/s00422-019-00792-y)

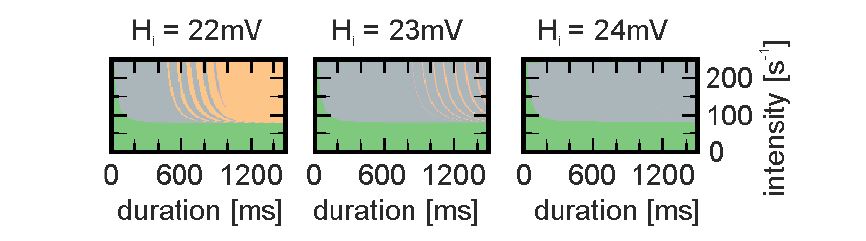

Supplement: Supplementary file 1 — Supplementary material 1 (JPEG 42 kb) [file 422_2019_792_MOESM1_ESM.jpg]

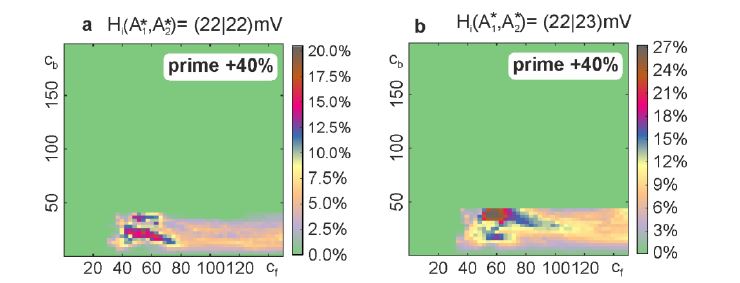

Supplement: Supplementary file 2 — Supplementary material 2 (JPEG 38 kb) [file 422_2019_792_MOESM2_ESM.jpg]
